# Epidemiological, Clinical, and Microbiological Characteristics in a Large Series of Patients Affected by *Dermacentor*-Borne-Necrosis-Erythema-Lymphadenopathy from a Unique Centre from Spain

**DOI:** 10.3390/pathogens11050528

**Published:** 2022-04-30

**Authors:** Sonia Santibáñez, Aránzazu Portillo, Valvanera Ibarra, Paula Santibáñez, Luís Metola, Concepción García-García, Ana M. Palomar, Cristina Cervera-Acedo, Jorge Alba, José R. Blanco, José A. Oteo

**Affiliations:** Center of Rickettsiosis and Arthropod-Borne Diseases (CRETAV), Infectious Diseases Department, San Pedro University Hospital-Center for Biomedical Research from La Rioja (CIBIR), 26006 Logroño, Spain; ssantibanez@riojasalud.es (S.S.); vibarra@riojasalud.es (V.I.); psantibanez@riojasalud.es (P.S.); lmetola@riojasalud.es (L.M.); cgarciag@riojasalud.es (C.G.-G.); ampalomar@riojasalud.es (A.M.P.); ccervera@riojasalud.es (C.C.-A.); jalbaf@riojasalud.es (J.A.); jrblanco@riojasalud.es (J.R.B.); jaoteo@riojasalud.es (J.A.O.)

**Keywords:** DEBONEL, *Dermacentor*-borne-necrosis-erythema-lymphadenopathy, *Dermacentor* *marginatus*, ‘*Candidatus* Rickettsia rioja’, *Rickettsia slovaca*, *Rickettsia raoultii*, *Rickettsia* sp. DmS1, Spain

## Abstract

During recent decades, a tick-borne rickettsial syndrome, characterized by eschar and painful lymphadenopathy after *Dermacentor marginatus*-bite, has been described as an emerging rickettsiosis in Europe. Our group named it DEBONEL (*Dermacentor*-borne-necrosis-erythema-lymphadenopathy), regarding the vector and the main infection signs. Other groups called it TIBOLA (tick-borne-lymphadenophathy) and, later, SENLAT (scalp-eschar-and-neck-lymphadenopathy-after-tick-bite), expanding, in the latter, the etiological spectrum to other pathogens. Objective: To investigate the etiology of DEBONEL agents in our area, and to compare their epidemiological/clinical/microbiological characteristics. During 2001–2020, 216 patients clinically diagnosed of DEBONEL (the largest series from one center) in La Rioja (northern Spain) were examined. *Rickettsia* spp. were amplified in 14/104 (13.46%) blood samples, 69/142 (48.59%) eschar swabs, 7/7 (100%) biopsies, and 71/71 (100%) *D. marginatus* from patients. For samples in which *Rickettsia* was undetected, no other microorganisms were found. ‘*Candidatus* Rickettsia rioja’, *Rickettsia slovaca*, *Rickettsia raoultii*, and *Rickettsia* DmS1 genotype were detected in 91, 66, 4, and 3 patients, respectively. DEBONEL should be considered in patients with clinical manifestations herein described in areas associated to *Dermacentor.* The most frequently involved agent in our environment is ‘*Ca.* R. rioja’. The finding of *Rickettsia* sp. DmS1 in ticks attached to DEBONEL patients suggests the implication of other rickettsia genotypes.

## 1. Introduction

During the past two decades, new tick-borne rickettsial diseases have been described in Europe [1]. One of these is known as DEBONEL/TIBOLA, acronyms of ‘*Dermacentor*-borne-necrosis-erythema-lymphadenopathy’ and ‘tick-borne lymphadenopathy’, respectively. DEBONEL/TIBOLA was described from several points of view; thus, compatible clinical cases were notified by Lakos et al. in Hungary [2], the microbiological approach was made by Raoult et al. in France [3], and the complete epidemiological, clinical and microbiological description was achieved in Spain and France [4,5,6,7]. Afterwards, several cases have been described in Bulgaria, Italy, Germany, Poland, Portugal, United Kingdom, France, and Spain [1,8,9,10,11,12,13,14,15,16,17,18,19,20]. *Dermacentor marginatus* is the main vector, although *Dermacentor reticulatus* has been also involved in France. After being bitten by a *Dermacentor* sp. tick, a high percentage of patients develop an inoculation eschar (point of necrosis) at the site of the tick-bite surrounded by an erythema and regional enlarged and painful lymphadenopathies. For these reasons, we proposed and defended the acronym DEBONEL since it makes reference to the main clinical features and to the involved tick genus. This fact has epidemiological implications because this tick genus is more active in the coldest months, when most cases appear. Regarding the etiological agents, *Rickettsia slovaca* was detected by polymerase chain reaction (PCR) in 1997 from a French patient with a scalp eschar and lymphadenopathy after a tick-bite in the Pyrenees mountains (France) [21]. Six years later, in 2003, the culture and isolation of *R. slovaca* from another French patient was reported, showing that *R. slovaca* is a human pathogen and an etiological agent of at least some patients affected with this syndrome [5]. In La Rioja (northern Spain), *R. slovaca* was also detected by PCR in ticks removed from DEBONEL patients [4,6,7,22,23]. In 2001, we achieved to amplify DNA corresponding to a new rickettsial genotype that we named ’*Candidatus* Rickettsia rioja’ (GenBank accession no. EF028201) in human blood and ticks removed from DEBONEL patients [24]. Moreover, *Rickettsia raoultii* [25]*,* initially named *Rickettsia* spp. RpA4, DnS14 and DnS28 [26,27], had been detected in ticks from DEBONEL patients [6,7,11]. In 2010, since the tick-bite is more frequently found on the scalp, Angelakis et al. proposed the name SENLAT (scalp-eschar-and-neck-lymphadenopathy-after-tick-bite) to describe this syndrome [28]. Nevertheless, this acronym is only useful when the tick-bite is on the scalp, and the term is also used in association with other infectious agents without referring to the arthropod vector that may belong to other tick genera, such as *Ixodes* sp. or *Rhipicephalus* sp. Apart from *R. slovaca*, *R. raoultii*, and ‘*Ca*. R. rioja’, the infectious agents include other *Rickettsia* spp. (*Rickettsia sibirica* subsp. *mongolitimonae* or *Rickettsia massiliae*) and non-*Rickettsia* microorganisms, like *Borrelia burgdorferi* sensu lato (s.l.), *Bartonella henselae*, *Francisella tularensis*, or *Coxiella burnetii* [28,29,30,31,32,33,34]. In this manuscript we will use our original term, DEBONEL, for naming the clinical picture of our patients since all of them fulfilled the criteria shown in the Section 4 and there is no consensual name. Herein, we describe the clinical and epidemiological characteristics of patients with DEBONEL caused by *R. slovaca*, ‘*Ca.* R. rioja’, and *R. raoultii* attended in the Department of Infectious Diseases at San Pedro University Hospital in La Rioja (SPUH) (Spain). The possible existence of epidemiological or clinical distinguishing features among DEBONEL patients infected with ‘*Ca*. R. rioja’, *R. slovaca*, or *R. raoultii* was also investigated. Moreover, we reported the first implication of the uncultured *Rickettsia* sp. DmS1 in three DEBONEL patients.

## 2. Results

Two hundred and sixteen out of 232 patients that met clinical and epidemiological inclusion’s criteria could be completely studied by microbiological methods and followed up. Seventy-one patients (32.87%) were attended with the tick attached or brought the tick removed by them or by sanitary personal. All the ticks were adults of *D. marginatus*, 63 females (88.73%) and 8 males (11.27%). The remaining patients 145 (67.12%) remembered being bitten by a large tick during the months in which *D. marginatus* is active, but they did not keep the arthropod.

**Figure 1:**
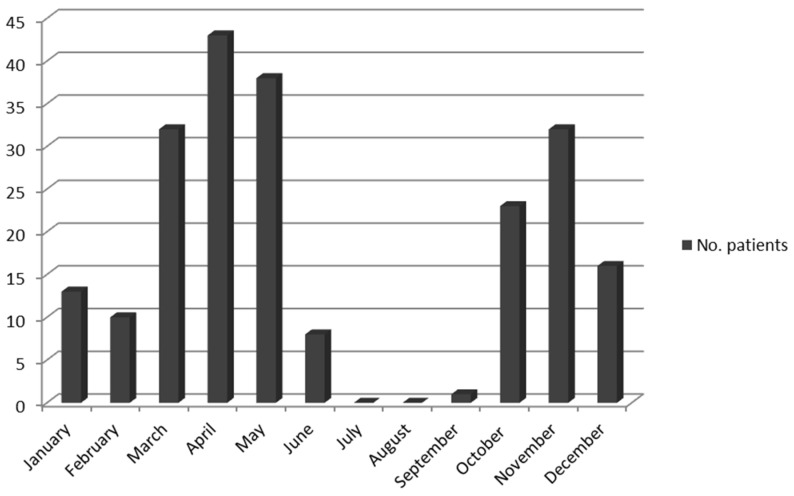
Monthly distribution of DEBONEL cases.

**Figure 2:**
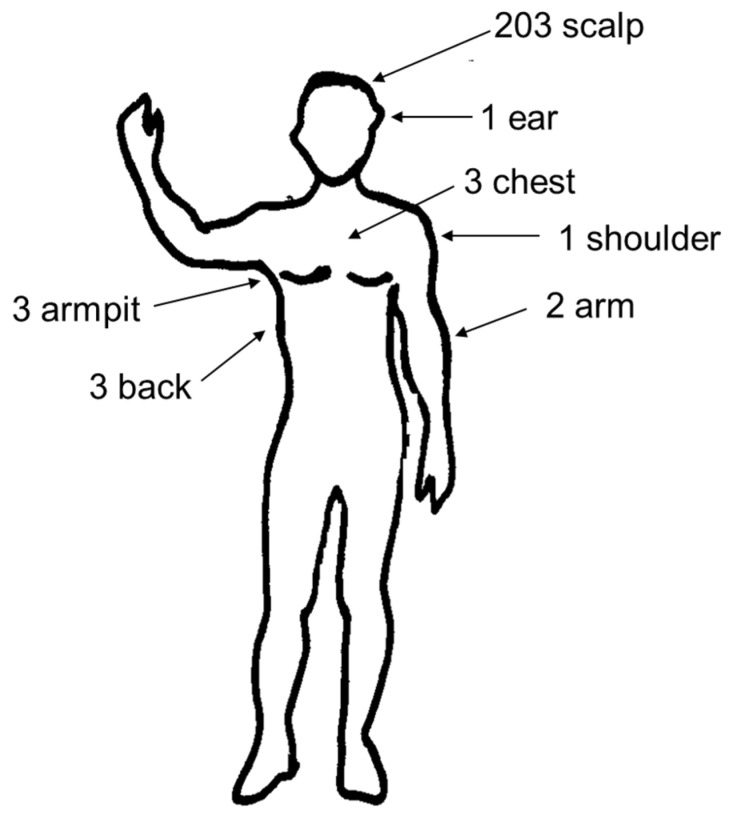
Location of skin lesions in DEBONEL patients.

Clinical and epidemiological data of the series are shown in Table 1. One hundred and forty-one patients (65.28%) were women and 75 (34.72%) were men. The mean age was 37.7 years (range 3–83) and the median age was 40. A total of 61 patients (28.24%) were younger than 15 years old. All patients were bitten mainly during the coldest months (from October to May), with a peak in November and in April–May (113 out of 216 cases), Figure 1. The incubation period varied from 1 to 15 days (mean: 5.61; median: 5). The tick-bite was located on the scalp in 203 patients (93.98%), and in 13 patients (6.02%) not on the scalp, including the back (3), armpits (3), arms (2), chest (3), ear (1), and shoulder (1), Figure 2. All patients with the inoculation lesion on the scalp shown local headache and multiple large and painful cervical lymphadenopathies. Facial local swelling was observed in 4 of them (1.97%). Furthermore, 76 patients (37.44%) developed alopecia at tick-bite site (0.5–2.0 cm diameter) that persisted after 3 months. Low grade fever (<38 °C axillary) was present in 71 patients (32.87%), and 8 patients (3.70%) had fever ≥38 °C. Diffuse macular rash was only observed in one patient (three macules in legs). All patients were treated with antibiotics. Doxycycline (100 mg/bid 14 days) was administered to 155 patients (71.76%), whereas 61 (28.24%) (59 children <15-year-old, a pregnant woman and a woman allergic to doxycycline) were treated with azithromycin (10 mg/Kg qd 5 days or 500 mg/qd 5 days). Improvement of the signs and symptoms were observed in all but one cases. This was a 15-year-old woman that worsened after 5-day-treatment with azithromycin. Later, she received a course of doxycycline and recovered. Fever disappeared 48 h after starting the treatment in all patients, and the painful lymphadenopathy improved in 1 week (5 to 15 days), although it was present during at least 3 or 4 weeks in most patients. 

### 2.1. Microbiological Tests

#### 2.1.1. Serological Assays

Evidence of recent infection (seroconversion or fourfold rise in titer) by a Spotted Fever Group (SFG) *Rickettsia*, was observed in 91 out of 109 patients with available paired sera (83.49%). It was detected during the first month in most cases, but seroconversion was delayed (in the second month) in 39 cases (24.84%).

#### 2.1.2. Molecular Methods (PCR)

PCR was performed in all 216 patients. A total of 14/104 blood samples (13.46%), 69/142 eschar swabs (48.59%), 7/7 biopsies (100%), and 71/71 *D. marginatus* (100%) were positive for *Rickettsia.* For those samples that yielded PCR negative results for *Rickettsia*, no other microorganisms were detected. 

The *ompA* and *ompB* genes sequences obtained from 8 blood samples, 38 eschar swabs, 4 biopsies, and 41 *D. marginatus* shown the highest similarity (99.4–100%) to ‘*Ca*. R. rioja’ (GenBank accession no. EF028201 and GQ404431, respectively). In three of them, positive PCR for *gltA* gene was obtained, and the nucleotide sequences shown the highest similarity (99.4%) with partial *gltA* gene from *Rickettsia* sp. DmS1 (GenBank accession no. AY129300). The *ompA*, *ompB* and *gltA* fragment genes amplified from 6 blood, 31 eschar swabs, 3 biopsies, and 26 *D. marginatus* were 100% identical to *R. slovaca* (GenBank accession no. CP002428.1). The *ompA* and *ompB* nucleotide sequences corresponding to the remaining four *D. marginatus* shown the highest similarity (99.3–99.8%) to *R. raoultii* strain Khabarovsk (GenBank accession no. CP010969).

**Figure 3:**
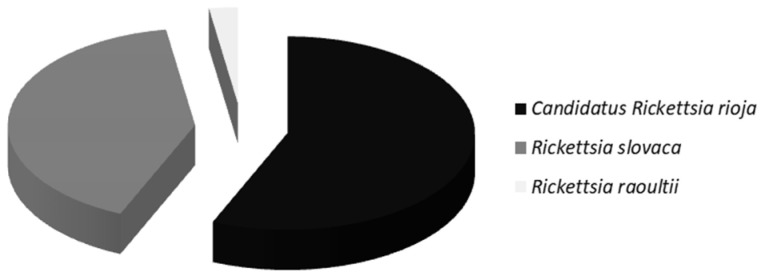
Etiological agents detected in DEBONEL cases.

In summary, diagnoses of infection by ‘*Ca*. R. rioja’ (91 cases), *R. slovaca* (66 cases), or *R. raoultii* (4 cases) were made in 161 out of 216 enrolled patients, Figure 3.

### 2.2. Epidemiological and Clinical Comparison of ‘Ca. R. rioja’, R. slovaca and R. raoultii Infections

#### 2.2.1. ‘*Ca*. R. rioja’ Infection

Ninety-one patients had evidence of *‘Ca*. R. rioja’ infection. More women (53.85%) than men were affected. Their mean and median age was 32.98 and 32.5 years (range 5–83), respectively; 38 of them (41.9%) were younger than 15 years old. All these patients were bitten during the coldest months (17 in March, 19 in April and May, 15 in November, 11 in October, 4 in February, 2 in December, 2 in January, and 2 in September). The incubation period ranged from 2 to 12 days (mean 5.43 and median 6 days). In 89 patients, the inoculation lesion was located on the scalp and two patients were bitten on the back. Two patients had fever that disappeared within 48h. after starting the antibiotic treatment. Fifty-eight patients received doxycycline (63.64%) and 33 azithromycin (36.26%). The painful lymphadenopathy improved during the first week for 39 out of 43 patients and for the remaining patients, during the following 15 days. Twenty-six out of 89 patients with the lesion on the scalp region (29.21%) developed persistent alopecia at the site of the tick-bite. 

Thirty-nine patients (81.25%) had evidence of recent infection by SFG *Rickettsia* based on indirect immunofluorescence assay (IFA) using *Rickettsia conorii* and *R. slovaca* antigens. Nine patients shown seroconversion during the second month.

#### 2.2.2. *R. slovaca* Infection

Sixty-six patients had evidence of *R. slovaca* infection. Forty-five were women (one was pregnant) and 21 were men. The mean and median age was 37.62 and 40.5 years (range 3–79), respectively; twenty-three of them (34.85%) were younger than 15 years old. Fifteen patients were bitten in April; 13 in November; 11 in May; 9 in March; 6 in January and October; and 2 in February, June, and December. The incubation period ranged from 2 to 12 days (mean and median 4.68 and 5 days). In 64 patients, the bite was located on the scalp, one patient was bitten on the arm and another one was bitten on the armpit. Only three patients had fever. All patients were treated with antibiotics, 43 with doxycycline (65.15%) and 24 with azithromycin (36.36%). In all cases, improvement of the signs and symptoms were observed. Fever disappeared 48h after the beginning of the treatment. The painful lymphadenopathy improved in one week for 27 out of 31 patients, and in 15 days for the remaining ones. Forty patients (62.50%) developed persistent alopecia at the site of the tick-bite (0.5–2 cm in diameter).

Evidence of recent infection by SFG *Rickettsia*-IFA, was observed in 28 patients (82.35%). The number of patients with reactivity against the two rickettsial antigens was similar. In all cases seroconversion was detected during the first month. In four cases, IgG antibody titers against *R. slovaca* were two serial dilutions higher than against *R. conorii*. 

#### 2.2.3. *R. raoultii* Infection

Four patients had evidence of *R. raoultii* infection. Two cases occurred in April, and one in May and in June. The incubation period was 5 days. In three patients, the bite was located on the scalp, while one patient was bitten on the chest. No patient had fever. In four cases, the painful lymphadenopathy improved in one week.

Evidence of recent infection with SFG *Rickettsia* was demonstrated by IFA in one patient. 

Comparison of data from human ‘*Ca*. R. rioja’, *R. slovaca*, and *R. raoultii* infections are shown in Table 1. Significant differences were detected in relation to the sex of the patients between those with DEBONEL caused by an unknown agent or by ‘*Ca*. R. rioja’. Besides, in terms of developing persistent alopecia significant differences were found between patients with ‘*Ca*. R. rioja’ infection and *R. slovaca* infection, and between those with *R. slovaca* infection and those with DEBONEL due to an unknown agent.

## 3. Discussion

In this report, we describe the epidemiological, clinical, and microbiological characteristics of 216 patients with DEBONEL (the largest series from a unique Center) observed over a 20-year period in La Rioja, a small region in the North of Spain, where this entity was first described by clinical and epidemiological observation and afterwards, as other groups, by microbiological techniques. Microbiological assays, and specifically PCR techniques, demonstrate that the etiological agents, when the clinical-epidemiological criteria are fulfilled, are ‘*Ca*. R. rioja’, followed by *R. slovaca* and *R*. *raoultii*. Furthermore, we have demonstrated that other *Rickettsia* genotypes, such as *Rickettsia* sp. DmS1, could be implicated. These facts are important since the empirical treatment made with doxycycline or azithromycin are effective against the involved agents [35,36]. As in other reports, there are clinical and epidemiological bases to diagnose DEBONEL [7,23]. It could be difficult to distinguish DEBONEL from tick-borne tularemia, since *Dermacentor* is involved in its transmission. In fact, in the 1990s a case of tularemia associated with *Dermacentor* was reported in our area [37], but the clinical picture was more severe. The studied area, La Rioja, is also endemic for Lyme borreliosis (LB) [38], but the clinical picture and the epidemiology should be enough to distinguish these two tick-borne diseases, although the activity of *Ixodes ricinus* (vector of LB) may sometimes overlap with *D. marginatus*. This last tick species typically inhabits steppes, meadows, and open forests. As in Central Europe, adult questing *D. marginatus* ticks start in late August and can last until May–June of the next year, including the winter months [39]. In addition, eschar inoculation is not present in LB, and the tick is usually unnoticed, or when noticed, it is smaller than the one that bites patients who develop DEBONEL. In our series, 100% of patients were aware of being bitten by a large tick. Searching in the literature, only four cases of tick-borne diseases related to *B. henselae* as agent of SENLAT have been published [28,34]. Moreover, we have studied a large number of *I. ricinus* and have not found *Bartonella* spp. (data not published). The possibility of a tick-borne rickettsiosis transmitted by *Rhipicephalus* spp. is, in our experience, easy to distinguish, since *R. conorii*, *R. sibirica* subsp. *mongolitimonae*, or *R. massiliae* and other possible *Rickettsia* transmitted by these ticks are associated with eschar, fever, malaise and other systemic manifestations. Besides, these ticks are more active in warm months. The same can be applied for the bite and illness caused by *Hyalomma* spp. Therefore, we recommend investigating the etiology when possible, although, in our environment, no patients with clinical picture of DEBONEL and negative microbiological studies for *Rickettsia* have shown the presence of *Francisella*, *Bartonella*, *Coxiella*, *Borrelia*, or other *Rickettsia* species different from those herein reported. In this series, we documented the etiology in 161 patients (74.53%) using molecular tools. Silva-Pinto et al. published in 2014 a review of 37 articles reporting TIBOLA/DEBONEL cases. The etiological agent was identified only in 149 out of the 537 (27.74%) cases of TIBOLA/DEBONEL, and, in most cases, it was *R. slovaca* [40]. In our study, ‘*Ca*. R. rioja’ was detected in 91 patients by PCR, *R. slovaca* in 66 patients and *R*. *raoultii* in four patients. These differences can be due to the geographical distribution of the agents. In addition, in our series, an uncultured rickettsial genotype DmS1 was amplified from three *D. marginatus* removed from patients. In 2003, *Rickettsia* sp. DmS1 was first detected in *D. marginatus* removed from game pigs [41], and, subsequently, it was detected in *D. marginatus* removed from asymptomatic patients from Eastern Spain [42]. Thus, to our knowledge, we describe here the first implication of this rickettsial genotype as human pathogen. In 55 out of 216 patients with identical clinical manifestations, the molecular methods did not allow us to achieve the identification of any SFG *Rickettsia*. These patients could either be infected by ‘*Ca* R. rioja’, *R. slovaca*, *R. raoultii*, *Rickettsia* sp. DmS1, or by another unidentified microorganism. Therefore, in our study we have not found any pathogens other than *Rickettsia* spp. from those associated with SENLAT. DEBONEL patients exhibit very typical and homogeneous epidemiological and clinical features. These characteristics were similar in patients infected by ‘*Ca* R. rioja’, *R. slovaca*, *R. raoultii*, and *Rickettsia* genotypes here involved, and in patients in whom etiological diagnosis could not be achieved. In all cases, the symptoms were mild, but 39% of patients presented sequelae as persistent alopecia at the site of the tick-bite. Regarding the most useful sample for the study of the etiological agent, we strongly recommend the use of eschar swabs that have allowed us to detect the etiological agent in half of the patients. It is also interesting to study the tick, since we have been able to identify rickettsial agents in 100% of the studied samples and they also allow the taxonomic identification of the vector [1,43]. The serological test, although sensitive for the diagnosis of rickettsiosis, does not allow an early microbiological diagnosis and it is not specific due to cross reactions demonstrated among *Rickettsia* spp. [44]. Throughout this study, we have incorporated real time-PCR assays, thus improving the sensitivity of the results. However, to know the etiologic agent, the best tool is the PCR and sequencing of the *ompA* gene [45]. In conclusion, in La Rioja, at least three different SFG *Rickettsia,* ‘*Ca*. R. rioja’, *R. slovaca*, and *R. raoultii,* besides the genotype DmS1, are responsible for the same disease that we named DEBONEL and other colleagues, TIBOLA. These agents are also involved as etiological agents of SENLAT. The terminology can lead to confusion and it would be time to look for a consensus name. TIBOLA does not reference to the eschar, which is the main clinical sign along with the lymphadenopathy. Since not all patients are bitten on the scalp, not all patients with the involved *Rickettsia* spp. can be included under the acronym SENLAT. 

Lastly, since DEBONEL is a prevalent rickettsiosis in the areas where *Dermacentor* spp. are distributed; its diagnosis should be considered in patients with the clinical manifestations herein described. 

## 4. Materials and Methods

### 4.1. Case Definition

The diagnosis of DEBONEL was made in patients who met, at least, the two following criteria: 1. A focus of necrosis (eschar) or a crusted lesion at the site of the tick attachment, surrounded by erythema and painful regional lymphadenopathy; and 2. Tick-bite by a *Dermacentor* sp. or a large tick during the period of maximum activity for *D. marginatus* (in La Rioja, mainly from the end of October to the beginning of May). Diagnosis of infections by *Rickettsia* spp. up to species level were based on PCR detection from clinical specimens, including the removed engorged ticks [1,6,43].

### 4.2. Patients and Samples

From January 2001 to December 2020, we prospectively studied all patients referred to SPUH (which serves all 314,000 inhabitants of the region) with suspicion of DEBONEL. Epidemiological data (age, sex, habits, contact with animals, rural/urban place of residence, etc.) were collected. Biological samples (EDTA-blood, sera, skin biopsies, eschar swabs and ticks removed from patients) were taken whenever possible, according to the moment of diagnoses. All these clinical samples are part of the “Zoonosis collection” registered in the National Registry of Biobanks of the Carlos III Health Institute (Reference: C.0006409), located in the Center of Rickettsiosis and Arthropod-Borne Diseases (CRETAV), Infectious Diseases Department, SPUH-Center for Biomedical Research from La Rioja (CIBIR), La Rioja, Spain. Patients were re-examined after one week and, whenever possible, at 4–12 weeks from the initial visit, depending on the severity of the clinical picture and to take sera samples in convalescent phases. We also evaluated the clinical response to the treatment (doxycycline or azithromycin for some children, pregnant women, and those patients allergic to doxycycline), according to our clinical experience and recommendations [35]. 

Approval of the regional ethics committee was obtained (Comité Ético de Investigación Clínica-Consejería de Sanidad de La Rioja, Ref. CEICLAR PI-37). Informed consent was obtained from all participants. All procedures were in accordance with the ethical standards of the research committee and with the 1964 Helsinki declaration and its later amendments.

### 4.3. Microbiological Tests

#### 4.3.1. Serological Assays

Whenever possible, acute-phase and convalescent sera (between 4–12 weeks), or at least acute sera, were tested by IFA for the presence of IgG antibodies against *R. conorii* [in-house (CRETAV) and/or commercial antigens (Vircell Microbiologists, Granada, Spain)] and *R. slovaca* [in-house (CRETAV) antigen]. Seroconversion or a fourfold rise in titer obtained from the late phase was considered evidence of recent infection by SFG *Rickettsia*.

#### 4.3.2. Molecular Methods (PCR)

The acute-phase sera, the EDTA-blood samples, and the eschar swabs, as well as all *D. marginatus* removed from patients, were analyzed by PCR assays. DNA was extracted using the DNeasy blood & tissue kit (QIAGEN, Hilden, Germany), according to the manufacturer’s recommendations. The presence of *Rickettsia* spp. in human samples and in ticks was determined by PCR assays targeting *ompA*, *ompB*, and *gltA* genes, as detailed in Table 2. Subsequently, *Bartonella* spp., *F. tularensis*, *B. burgdorferi* s.l., and *C. burnetii* were screened by PCR assays (Table 3) when negative results for SFG *Rickettsia* were obtained and in selected samples. Quality controls included both positive ones, grown in Vero cells [*R. conorii* Malish # 7 (up to year 2009), or *Rickettsia amblyommatis* (during 2010–2020) from CRETAV collection, and negative controls (containing sterile water instead of template DNA) that were extracted and tested in parallel with all specimens. Other positive controls, such as *B. henselae* DNA extracted from a cat flea —*Ctenocephalides felis*— from La Rioja (Spain), double stranded synthetic gBlock of *F. tularensis* DNA (Integrated DNA Technologies, Coralville, IA, USA), *Borrelia spielmanii* DNA (kindly provided by Dr. Volker Fingerle, German National Reference Centre for Borrelia, Germany), and commercially available Amplirun® *C. burnetii* DNA control (Vircell Microbiologists, Granada, Spain) were included in PCR assays. Sequencing reactions were carried out and results were analyzed through GenBank database using BLAST utility (National Center for Biotechnology Information; available from: URL; http://www.ncbi.nlm.nih.gov, accessed on 29 April 2022).

## Figures and Tables

**Table 1:** Comparison of clinical and epidemiological data from human ‘*Candidatus* Rickettsia rioja’, *Rickettsia slovaca* and *Rickettsia raoultii* infection.

Clinical and Epidemiological Data	*‘Ca*. R. rioja’ Infection (n:91)	*R. slovaca* Infection (n:66)	*R. raoultii* Infection (n:4)	Patients with DEBONEL by Unknown Agent (n:55)	*p* Value	Total Patients (n:216)
**Sex (female)**	49/91 (53.85%)	45/66 (68.18%)	3/4 (75.00%)	44/55 (80.00%) **	0.008	141
**Mean age (years)**	32.98 ± 2.42	37.62 ± 3.09	27.00 ± 12.27	39.13 ± 2.80	0.325	37.7
**IP (days)**	5.43 ± 0.31	4.68 ± 0.28	5.00 ± 0.41	5.78 ± 0.40	0.161	5.61
**Low grade fever ^1^**	34/91 (37.36%)	19/66 (28.79%)	2/4 (50.00%)	16/55 (29.09%)	0.505	71 (32.87%)
**Fever ^2^**	2/91 (2.20%)	3/66 (4.55%)	0/4 (0.00%)	3/55 (5.45%)	0.653	8 (3.70%)
**Persistent alopecia ^3^**	26/89 (29.21%)	40/64 (62.50%) ***	1/3 (33.33%)	9/47 (19.15%) ^###^	<0.001	76/203 (37.44%)
**Evidence of recent infection by IFA (seroconversion or fourfold rise in titer)**	39/48 (81.25%)	28/34 (82.35%)	1/1 (100.00%)	23/26 (88.46%)	0.798	91/109 (83.49%)

Qualitative variables are represented in percentage while quantitative variables are represented as mean ± standard error mean. The *p* value refers to the comparison between four groups. Asterisks indicate statistically significant differences with respect to ‘*Ca*. R. rioja’ group, while hashtags indicate statistically significant differences with respect to *R. slovaca* (** *p* < 0.01 vs. ‘*Ca*. R. rioja’, *** *p* < 0.001 vs. ‘*Ca*. R. rioja’ and ### *p* < 0.001 vs. *R. slovaca*). ‘*Ca*. R. Rioja’: ‘*Candidatus* Rickettsia rioja’; n: number; *R.*: *Rickettsia*; DEBONEL: *Dermacentor*- borne-necrosis-erythema-lymphadenopathy; IP: Incubation period; ^1^ Low grade fever: <38 °C; ^2^ Fever > 38 °C; ^3^ Persistent alopecia: The patient developed persistent alopecia at the site of the tick-bite (0.5–2 cm in diameter); IFA: Immunofluorescence assay.

**Table 2:** Primer pairs used for amplification of rickettsial genes.

Gene	Primers	Primer Sequence (5′→3′)	Fragment Size (bp)	Tm (°C)	Reference
*gltA* (nested)	RpCS.877p	GGGGGCCTGCTCACGGCGG	381	48	[46,47]
	RpCS.1258n	ATTGCAAAAAGTACAGTGAACA			
	RpCS.896p	GGCTAATGAAGCAGTGATAA	337	54	
	RpCS.1233n	ATTGCAAAAAGTACAGTGAACA			
*ompA* (semi nested)	Rr190.70p	ATGGCGAATATTTCTCCAAAA	631	46	[46,48]
	Rr190.701n	GTTCCGTTAATGGCAGCATCT			
	Rr190.70p	ATGGCGAATATTTCTCCAAAA	532	48	
	Rr190.602n	AGTGCAGCATTCGCTCCCCCT			
*ompB* (nested)	rompB OF	GTAACCGGAAGTAATCGTTTCGTAA	511	54	[47]
	rompB OR	GCTTTATAACCAGCTAAACCACC			
	rompB SFG IF	GTTTAATACGTGCTGCTAACCAA	420	56	
	rompB SFG/TG IR	GGTTTGGCCCATATACCATAAG			

**Table 3:** PCR primer pairs used for screening of *Bartonella* spp., *Francisella tularensis*, *Borrelia burgdorferi* sensu lato, and *Coxiella burnetii*.

Bacteria	Target Gene	Primer Sequence (5′→3′)	Fragment Size (bp)	Tm (°C)	Reference
*Bartonella* spp.	*rpoB*	CGCATTGGCTTACTTCGTATG GTAGACTGATTAGAACGCTG	825	53	[49]
*Francisella tularensis*	17 KDa lipoprotein	ATGGCGAGTGATACTGCTTG GCATCATCAGAGCCACCTAA	250	56	[50]
*Borrelia burgdorferi* sensu lato	*Flagellin* (nested)	AARGAATTGGCAGTTCAATC GCATTTTCWATTTTAGCAAGTGATG	497	52	[51]
		ACATATTCAGATGCAGACAGAGGTTCTA GAAGGTGCTGTAGCAGGTGCTGGCTGT	389	55	[51,52]
*Coxiella burnetii*	IS*1111*	TATGTATCCACCGTAGCCAGTC CCCAACAACACCTCCTTATTC	685	48	[53]

W: A/T; R: A/G.

## Data Availability

Not applicable.
